# Retinitis Pigmentosa Treatment with Western Medicine and Traditional Chinese Medicine Therapies

**DOI:** 10.1155/2015/421269

**Published:** 2015-06-01

**Authors:** Jian Xu, Qinghua Peng

**Affiliations:** ^1^Post-Graduate School, Hunan University of Traditional Chinese Medicine, Changsha, Hunan 410208, China; ^2^Department of Ophthalmology, the First Affiliated Hospital of Hunan University of Traditional Chinese Medicine, Changsha, Hunan 410007, China

## Abstract

Current management of retinitis pigmentosa (RP) includes an attempt at slowing down the degenerative process through therapies that use either Western or traditional Chinese medicine (TCM). Novel therapies in Western medicine (WM) include use of tailor-made gene therapy, transplantation of stem cells, or neuroprotection treatment. TCM treatment includes two major approaches. These are orally applied herbal decoctions and acupuncture. In fact, all TCM treatments are based on the differentiation of a symptom-complex, which is the characteristic essence of TCM. Thus, diagnosed RP may be treated via the liver, the kidney, and the spleen. The principle behind these treatments is to invigorate the blood and brighten the eyes by toning up the liver and the kidney. Also treatments to cope with deficiencies in the two concepts that are unique and fundamental to TCM are considered: Qi or “vital energy” and Yin and Yang or the harmony of all the opposite elements and forces that make up existence. In particular, the Qi deficiency that results from blood stasis is addressed in these treatments. This paper also puts forward the existing problems and the prospect of the future development on integrating TCM with WM.

## 1. Introduction

Retinitis pigmentosa (RP) is a group of inherited degenerative retinal diseases, involving progressive degeneration of the retina, typically starting in the midperiphery and advancing toward the macula and fovea [1]. It is associated with night blindness, progressive peripheral visual field loss followed by reduction in central vision, and abnormalities in the electroretinogram (ERG). The prevalence of RP is reported to be 1 in 3000–7000 individuals worldwide [2], with more than 1.5 million patients who suffer from progressive visual deterioration with this disorder [3]. Most RP patients suffering from low-vision or blindness are often severely disabled or legally blind by the end of the second, third, or fourth decade of life. For that reason, it is important that our work focuses on therapy. Over the last few decades, several therapies have been devised for the treatment of RP with gene therapy, stem cell therapy [4], neuroprotection therapy, and TCM treatment, included amongst them.

## 2. Western Medicine Therapies

### 2.1. Stem Cell Therapy

Various types of stem cells have been isolated from a variety of tissues including preimplantation embryos, fetuses, birth-associated tissues, and adult organs. Based on the source, these cells can be broadly classified into embryo-derived stem cells (ESCs) and adult tissue-derived stem cells. And based on biochemical and genomic markers, these stem cells can be broadly classified into embryonic stem cells (ESCs), mesenchymal stem cells (MSCs), hematopoietic stem cells (HPSCs), and induced pluripotent stem cells (IPSCs). Stem cell therapy is a novel approach for vision restitution in retinitis pigmentosa [5]. Transplantation of stem cells that can be stimulated to become replacement photoreceptors and be supportive of outer retina cells can theoretically lead to treatments that restore visual function [6].

In recent years, stem cell transplantation therapy in RP has made progress. The mechanisms of stem cells therapy for this disease include the following.

1. Cell replacement: transplanted stem cells can differentiate into retinal cells and integrate into the retina of patients, and the differentiated stem cells replace the apoptotic or injured retinal cells [7].
2. Nutritional support: the function of transplanted cells is to release diffusible factors and nutritional substances which act as a local cell delivery system for trophic factors and can promote photoreceptor cell survival [8–10].
3. Protection of the retinal blood vessels and cones: bone marrow derived stem cells contain endothelial precursors. Through significant upregulation of many antiapoptotic genes, these stem cells can rescue retinal blood vessels that would ordinarily degenerate completely [11, 12]. Researchers have observed that in humans, either the patient's or a normal person's bone marrow cells may provide potential cone neuroprotection to preserve central vision [13].
4. Promotion synaptic connections: many studies have shown donor cells taken from developing mouse retina at a time coincident with the peak of rod genesis can blend in with cells in a normal adult or a degenerating mouse retina and subsequently build synaptic connections with the remaining retinal cells and, thus, effectively improve visual function [14]. Despite the recent progress made by stem cell transplantation therapy as a treatment for retinal degeneration, many challenges remain. Firstly, the problem of low rate of stem cell survival and migration needs to be resolved. Secondly, despite the retina being physically isolated from the immune system, the immune response that is triggered when stem cells are transplanted into the subretinal space during therapy and hampers their survival cannot be ignored. Thirdly, there are some biosafety issues. For example, the formation of tumors by transplants cannot be ruled out and, therefore, the therapeutic suitability of the stem cells transplanted which include factors such as the age of the patient cannot be ruled out.

### 2.2. Gene Therapy

The mechanism in gene therapy is the transfer of a therapeutic gene by use of viral or nonviral vectors and requires genetic modification of the ocular cells to produce its therapeutic effect. There are two methods to replace or correct abnormal genes: (1) gene augmentation therapies, where a normal gene is inserted into the genome to replace nonviable or diseased genes using a carrier vector; (2) gene silencing therapies, in which the expression of the mutated gene is inhibited by use of ribozyme or RNA interference [15].

Successful augmentation therapies are dependent on efficient transduction of the target cell by Adeno-associated virus (AAV) and sustained expression of the recombinant virus at a sufficient level. To date, more than 30 patients have so far received the gene therapy, ranging in follow-up from 90 days to 1.5 years and no major side effects have been reported [16, 17]. Two approaches have been proposed to silence the abnormal gene: ribozymes and RNAinterference (RNAi). RNAinterference (RNAi) knockdown is an efficacious therapeutic strategy for silencing genes causative for dominant retinal dystrophies [18]. Some studies showed that allele-specific or non-allele-specific knockdown of a dominant GCAP1 mutant can ameliorate photoreceptor dystrophy in dominant RP and cone-rod dystrophy mouse models caused by GCAP1 mutations [18]. However, it may be difficult to judge which model is most relevant to a specific condition in humans. Some of the mutations between humans and animals are not similar. The mutation and the phenotype in the animal model must be viewed with some degree of caution. It cannot therefore be assumed that these are truly representative of the disease that is occurring in the patient until the phenotypes are critically examined using the same criteria. Translational clinical research initiatives are finally offering hope to relatives and patients with RP, but the safety of these techniques has yet to be established in large animal and human experiments.

### 2.3. Neuroprotection Therapy

Neuroprotection provides a sympathetic environment to prolong the viability of the photoreceptors by their effect on the secondary biochemical pathways. It is a therapeutic strategy which can be achieved either by delivering neurotrophic growth factors, on the one hand, or inhibiting proapoptotic pathways on the other. It can also be delivered by the implementation of viability factors such as the rod-derived cone viability factor for the treatment of retinal neurodegenerative disease that is independent of the etiology of the degeneration. A number of neurotrophic growth factors that slow photoreceptor death in animal models have been identified: basic fibroblast-derived growth factor (bFGF), brain-derived neurotrophic factor (BDNF), cardiotrophin-1, nerve growth factor (NGF), fibroblast growth factor (FGF), and CNTF.

The final common pathway of all types of RP is photoreceptor cell death. Leonard et al. [19] reported that the X-linked inhibitor of apoptosis protein (XIAP) was thought to be the most potent member to promote cell preservation. They used Adeno-associated virus (AAV) mediated delivery of XIAP to study its neuroprotective effect. Their results showed that XIAP treated eyes of homozygous albino transgenic rats had significantly preserved outer nuclear layers than their contralateral untreated counterparts. Recent evidence [20, 21] has highlighted the importance of calpain activation for both photoreceptor cell death and survival. The authors of these studies have proposed the use of highly specific calpain inhibitors to prevent or delay RP. Recent evidence derived from studies on rod-derived cone viability factor (RdCVF) protein injections in a type of rhodopsin mutation, in the P23H rat, showed that this induced an increase in cone cell number. This suggests that RdCVF is a promising therapeutic option for saving rods [22]. Another new study [3] has shown that decreased cellular ATP levels may result in the pathology of this eye disease and perhaps also in RP and other similar neurodegenerative diseases. Therefore, neuroprotection may prevent or forestall the progression of such incurable eye diseases that ultimately lead to blindness. Ikeda et al. [3] described small compounds (Kyoto University Substances, KUSs) that were developed to inhibit the ATPase activity of valosin-containing protein (VCP), which is the most abundant soluble ATPase in the cell. The authors showed that KUSs, as well as exogenous ATP or ATP-producing compounds, suppressed endoplasmic reticulum stress and demonstrably protected various types of cultured cells including retinal ones from death. KUSs prevented photoreceptor cell death and preserved visual function in rd10, a mouse model of RP.

## 3. Traditional Chinese Medicine (TCM) Therapies

### 3.1. TCM Pathogenesis of RP

TCM therapy is based mainly on the practice of Chinese medicine and is constantly enriched and developed by practical experience. In ancient times, Chinese people discovered that certain foods reduced or eliminated certain diseases. This became the basis of Chinese herbal medicine. It is thought that ancient peoples discovered that fomenting a painful area of the body with the warmth of a fire or the use of warm leather or bark bags or hot stones or sand eliminated the discomfort caused by pain. While using stone as a production tool, people discovered that after a certain part of the body being stabbed, the pain in another part could be relieved, thereby creating treatment methods of using stone and bone needles, which was developed into acupuncture and therefore the formation of meridian.

Retinitis pigmentosa (RP) belongs to the high-altitude wind sparrow's vision category in TCM. High-altitude wind sparrow's vision was described in the book* Taiping-sengxian Prescriptions* in 992 AD for the first time. This classical work recorded more than 16,800 prescriptions and was descended to later generations popularly. Treatment determination based on syndrome differentiation is an essential principle in TCM in understanding and treating disease. It is a specific research and treatment method of disease in TCM and also one of the essential characteristics of TCM. TCM believes that the congenital deficiency with debilitation of the life gate fire is the main reason that causes RP. Other pathogenic factors of RP in TCM include liver and kidney deficiency with essence and blood insufficiency, or spleen and stomach deficiency with Qi and blood insufficiency. Finally, these elements can result in blood stagnation and vessel insufficiency, leading to a loss of nourishment to the eyes, and these will whittle down the spirit light of the pupil, narrow of the visual field, and nocturnal blindness. In TCM, this condition is referred to as high-altitude wind internal obstruction or high-altitude wind sparrow's vision. In recent years, many TCM ophthalmologists conducted further study of the pathogenesis of this disease. For example, Peng [23] proposed and demonstrated blood stasis in deficient pattern type of pathogenesis in patients with retinitis pigmentosa. They put forward that in the treatment of this disease, some additional Chinese medicines which can remove the blood stasis and promote the blood circulation need to be used on the basis of ameliorating deficient pattern and, by doing so, may achieve more satisfactory results [24]. This study gives some new insights on Chinese medicine treatment for RP. However, the main hurdle remaining in the traditional Chinese medicine (TCM) theory is that the success of these treatments depends on proper symptom-complex differentiation, which may also include the stage of the disease, the patient's age, systemic and environmental factors, and so on.

Orally applied herbal decoctions and acupuncture treatment have a role in relieving symptoms and improving visual function in patients.

### 3.2. Oral Herbal Decoctions for RP Treatment

All TCM treatments are differentiated on the symptom-complex seen. This is the essence of TCM therapy. High-altitude wind sparrow's vision can be treated from the liver, the kidney, or the spleen. TCM differentiates the pathogenesis of this disease into four different syndromes, including deficiency of the liver-Yin and kidney-Yin, deficiency of the spleen and Qi, insufficiency of the kidney-Yang, and deficiency of the Qi and blood stasis. Among these multifaceted symptom complexes, deficiency of the liver-Yin and kidney-Yin is the most common clinical pattern observed. Different syndromes are prescribed very different treatments. For example, deficiency of the liver-Yin and kidney-Yin is treated by modified Ming Mu Di Huang decoction, which nourishes and tones the liver and the kidney and leads to invigorating the blood and brightening the eyes. In this formula of modified Ming Mu Di Huang decoction, the Chinese herbal drugs, Shudihuang, Danggui, Wuweizi, and Gouqizi, work on supplementing liver and kidney. The Chinese herbal drugs, Danpi, Danshen, Yemingsha, and Chongweizi, can also clear heat and invigorate blood. Chinese herbal drugs Shan Zhu Yu and Shen Di Huang reinforce the kidney, which may improve eyesight by replenishing vital essence. The formula achieves the purpose of seeking Yang within Yin, bringing true Yang back to its origin and unblocking the vessels and collaterals. So the actions of this formula are nourishing and tonifying liver and kidney. And they can invigorate blood and brighten the eyes. A recent study [25] in China reported that using TCM treatment on RP resulted in a satisfactory clinical curative affect, and it was worth publicizing. In this study, researchers observed 83 eyes of 42 patients whose TCM diagnoses were high-altitude wind sparrow's vision (deficiency of the liver-Yin and kidney-Yin). After treatment by modified Ming Mu Di Huang decoction, the visual acuity and visual fields in these cases had obvious improvement. Many studies demonstrate that the modified Ming Mu Di Huang decoction may protect apoptosis of photoreceptor cells in retinal degeneration. So it stabilizes the symptoms and delays the progression of RP. The blood moving medicinals include Dang Gui and Dan San that enhance the effect of nourishing and invigorating blood. And thus, they make an impact on the preserving the vision and prevent optic atrophy or blindness.

### 3.3. Acupuncture for RP Treatment

Acupuncture has been applied as a therapeutic medical technique in China since at least 2,000 years ago, when stone knives and other sharp instruments were used. The term itself is derived from Latin “acus” meaning needles and “punctura” meaning puncture. In this form of treatment, some diseases of the body can be treated by puncturing the points on the body surface to regulate the meridians, Zang-Fu organs, and the circulation of Qi and blood. Acupuncture treatment in RP cases often takes the acupoints such as BL1 (Jing Ming), EX-HN7 (Qiu Hou), GB20 (Feng Chi), LB18 (Gan Shu), LB20 (Pi Shu), ST36 (Zu San Li), and SP6 (San Yin Jiao). The therapist selects 1-2 local points along with two distal acupoints each time. The points should be made with the reinforcing method of needle, which is thought to invigorate the body's healthy Qi and to strengthen weakened physiological function. The filiform needle should be kept for 20–30 minutes in place insertion. For patients who are chronically Yang deficient, one should apply the moxibustion on the distal points or use both needling with moxa. Moxibustion is a therapy in which burning moxa is used to produce a heat stimulation to the human body. It affects the function of the meridians and points to treat or prevent diseases.

Acupuncture may improve the activities and speed of the rod and cone cells of retina, enhance the neural networks and biological activity of the retina cells, and improve the inner circulation, the metabolic activities of retinal epithelium-photoreceptors complexes, and the damages to visual function. Ma et al. [26] used acupuncture dialectical therapy in 15 cases of RP and observed visual acuity, visual field, ERG, and other indicators. The clinical study showed that after treatment, the vision acuity, the visual field distribution, and the ERG-b wave had been improved. The total effective rate was 86.7% and the differences were statistically significant.

However, one problem of acupuncture therapy is that there is no uniform standard and parameters on acupuncture point selection, needling techniques, depth and angle of piercing, gas-getting status, needle retention time, and stimulation methods.

### 3.4. Common Clinical Patterns and Clinical Treatments in TCM

Some common clinical patterns and clinical treatments in traditional Chinese medicine is summarized in Table 1.

## 4. Challenges and Outlook

There are many topics in clinical studies on RP. The current approaches against RP include the Chinese herbal medicine, acupuncture, moxibustion, gene therapy, neuroprotection therapy, neurotrophic growth factors, antiapoptotic agents, ribozyme therapy, RNAi, retinal transplantation, dietary supplementation, retinal prostheses, stem cell therapy, and so on. However, due to the complexity of RP pathogenesis, multiple risk factors, long cycle of RP prevention and control, and the overall prognosis of severe RP remains dismal. There are some obstacles: the success of these treatments depends on proper patient selection; how to successfully translate new therapies in the animal models of the disease; how to effective delivery of the therapy, both genetic material and other neurotrophic factors, and stem cell to the target tissue have been a formidable task. Worldwide researches with numerous samples are expected.

From the long-term perspective, delaying the occurrence and progression of RP and establishing an efficient and practical prevention and control system is the focus of the future RP research in the world. TCM may be able to play an important role in this. Possible future research direction of integrating TCM with western medicine may include (a) TCM treatment of RP by regulating stem cells and (b) TCM treatment of RP by regulating microglia.

In addition, for complicated life phenomenon, both metabonomics and pharmacometabonomics take an organic conception of the human body, which conforms to the way of thinking of traditional Chinese medicine (TCM). The application of metabonomics and pharmacometabonomics in the TCM treatment in RP can deepen the evaluation of the therapeutic effects of TCM in RP through the study of intrinsic quality of TCM syndrome and the treatment by differentiation of syndrome. The research about integration of TCM with modern biological science and technology in RP may provide a new space for the development of therapy against RP, and these treatments would fill an enormous therapeutic gap that we have right now.

## Figures and Tables

**Table 1:** Common clinical patterns and clinical treatments in TCM.

	Kidney-Yang deficiency	Liver- and Kidney-Yin deficiency	Spleen Qi deficiency
Tongue body	Pale with a white coating	Pink with a thin coating	Pale with teeth marks and a thin white coating

Pulse	Deep and weak	Thready	Thready and weak

Principles	Warm and tonify kidney-yang, invigorate blood, and brighten the eyes	Nourish and tonify liver and kidney, invigorate blood, and brighten the eyes	Supplement the spleen and benefit Qi, invigorate blood, and brighten the eyes

Formula	Modified You Gui Wan Zhi Fu Zi 6 g Rou Gui 6 g Shu Di Huang 15 g Shan Zhu Yu 10 g Sang Shen Zi 12 g Gou Qi Zi 15 g Huai Shan Yao 10 g Dang Gui 10 g Tu Si Zi 15 g Rou Cong Rong 10 g Chuan Xiong 10 g Niu Xi 15 g	Modified Liu Wei Di Huang Wan Shudihuang 15 g Shan Zhu Yu 15 g Huai Shan Yao 15 g Mu Dan Pi 10 g Fu Ling 10 g Ze Xie 10 g Dan Shen 10 g Niu Xi 10 g Chuan Xiong 10 g Gan Cao 6 g	Modified Bu Zhong Yi Qi Tang Chai Hu 10 g Huang Qi 15 g Dang Shen 10 g Bai Zhu 10 g Dang Gui 10 g Ge Gen 20 g Hong Hua 3 g Man Jing Zi 10 g Bai Ji Li 10 g Dan Shen 12 g Ye Ming Sha 12 g Cang Zhu 10 g Gan Cao 5 g

Acupuncture (main points)	BL1 (Jing Ming) ST1 (Cheng Qi) EX-HN7 (Qiu Hou) GB20 (Feng Chi) BL18 (Gan Shu) BL23 (Shen Shu) ST36 (Zu San Li) GB37 (Guang Ming) SP6 (San Yin Jiao)	GB20 (Feng Chi) EX-HN13 (Yi Ming) EX-HN7 (Qiu Hou) BL2 (Cuan Zhu) ST2 (Si Bai) SI6 (Yang Lao) LI4 (He Gu) SP6 (San Yin Jiao) LR3 (Tai Chong) BL18 (Gan Shu) BL23 (Shen Shu) KI3 (Tai Xi)	BL17 (Ge Shu) BL20 (Pi Shu) GB20 (Feng Chi) EX-HN13 (Yi Ming) EX-HN7 (Qiu Hou) BL2 (Cuan Zhu) ST2 (Si Bai) SI6 (Yang Lao) LI4 (He Gu) SP6 (San Yin Jiao) LR3 (Tai Chong)
